# Salmonid Alphavirus Subtype 3 Induces Prolonged Local B Cell Responses in Atlantic Salmon (*Salmo salar*) After Intraperitoneal Infection

**DOI:** 10.3389/fimmu.2020.01682

**Published:** 2020-09-10

**Authors:** Shiferaw Jenberie, Ma. Michelle D. Peñaranda, Hanna L. Thim, Morten Bay Styrvold, Guro Strandskog, Jorunn B. Jørgensen, Ingvill Jensen

**Affiliations:** Norwegian College of Fishery Science, Faculty of Biosciences, Fisheries and Economics, The Arctic University of Norway, Tromsø, Norway

**Keywords:** antibody secreting cells, Atlantic salmon, B cells, peritoneal cavity, Salmonid alphavirus, teleost

## Abstract

B cell responses are a crucial part of the adaptive immune response to viral infection. Infection by salmonid alphavirus subtype 3 (SAV3) causes pancreas disease (PD) in Atlantic salmon (*Salmo salar*) and is a serious concern to the aquaculture industry. In this study, we have used intraperitoneal (IP) infection with SAV3 as a model to characterize local B cell responses in the peritoneal cavity (PerC) and systemic immune tissues (head kidney/spleen). Intraperitoneal administration of vaccines is common in Atlantic salmon and understanding more about the local PerC B cell response is fundamental. Intraperitoneal SAV3 infection clearly induced PerC B cell responses as assessed by increased frequency of IgM^+^ B cells and total IgM secreting cells (ASC). These PerC responses were prolonged up to nine weeks post-infection and positively correlated to the anti-SAV3 E2 and to neutralizing antibody responses in serum. For the systemic immune sites, virus-induced changes in B cell responses were more modest or decreased compared to controls in the same period. Collectively, data reported herein indicated that PerC could serve as a peripheral immunological site by providing a niche for prolonged maintenance of the ASC response in Atlantic salmon.

## Introduction

Adaptive humoral immunity is an essential component of the defense against viral infections. Teleost fish are one of the oldest living groups of organisms possessing elements of the adaptive immune system as described in mammals. However, some major differences are present making this species a useful comparative model of lower vertebrate immunology [reviewed in Ref. (1)]. While lymph nodes and bone marrow are lacking, the major systemic lymphoid tissues in teleosts involved in B cell generation and activation are the anterior kidney (head kidney, HK) and spleen. Ig (immunoglobulin) class switching is absent in teleosts and hence, they rely on un-switched IgM responses with limited affinity maturation upon repeated immune challenges [reviewed in Ref. (1)]. IgM is the principal systemic Ig, which is most abundant in serum and is co-expressed with IgD on the surface of teleost B cells (2). IgT is the predominant Ig at mucosal surfaces and is secreted by a separate B cell lineage uniquely expressing this isotype (3). Recent data from rainbow trout (*Oncorhynchus mykiss*) support the view that teleost B cells resemble the mammalian subset of B-1 B cells (4). However, comprehensive knowledge about B cell subsets and corresponding markers are insufficient in teleosts.

Vaccination can be highly protective in teleost fish, and multicomponent vaccines have contributed tremendously to disease prophylaxis in the aquaculture industry (5). However, viral diseases continue to be a major challenge to fish welfare, calling the efficacy of viral vaccines into question. The majority of vaccines in use in aquaculture are delivered by intraperitoneal (IP) injection. In this regard, understanding more about the local B cell response in the peritoneal cavity (PerC) is fundamental. It is well-known that the PerC of teleost fish holds a varying proportion of resident myeloid and lymphoid cells (6–11). Intraperitoneal administration of various stimuli leads to immune cell mobilization in the teleost PerC characterized by an increase of highly phagocytic neutrophil-and macrophage-like cells within hours and days after the insult (12–14). The total number of PerC IgM^+^ B cells is also influenced by IP stimulation and in rainbow trout these cells represented more than two thirds of all PerC cells 72 h after IP bacterial immunization (7). Recent studies have also shown activation and differentiation of B cells into plasmablast-like cells in the PerC, thus suggesting the existence of a niche that supports B cell differentiation (15–17). In teleost PerC, adipose tissue may function as a peripheral immune site by retaining antigens and thereby contributing to the overall B cell response (15). However, there is currently limited knowledge about how teleost PerC B cells respond to viral infection and how the interplay between the local PerC response and systemic lymphoid tissues affects this.

Salmonid alphavirus (SAV) is the etiological agent of pancreas disease (PD), a serious disease affecting farmed Atlantic salmon (*Salmo salar*) and rainbow trout [reviewed in Ref. (18)]. Salmonid alphavirus is a member of the Togaviridae family and is an enveloped single-stranded positive-sense RNA virus. Six genotypes of SAV have been identified (SAV 1–6) (19), with SAV3 being one of the subtypes causing major problems in Atlantic salmon aquaculture. Atlantic salmon can acquire long-term protection against PD after recovering from the disease (20). Salmonid alphavirus infection induces neutralizing antibody (Ab) responses (21–23) and passive immunization experiments indicate that these are involved in promoting protective immunity (24). There are commercial vaccines against PD in use based on inactivated SAV, which are administered by IP injection, despite this, disease outbreaks continue to occur in vaccinated fish [reviewed in Ref. (18)]. Here, we have used SAV3 infection by IP injection as a relevant model to study local versus systemic B cell responses in Atlantic salmon over a period of nine weeks. Notably, the findings demonstrate a prolonged presence of virus-induced IgM^+^ cells and IgM secreting cells (ASC) in Atlantic salmon PerC. For the systemic immune sites, virus-induced changes in B cell responses were more modest or decreased. Collectively, the presented work indicate that the PerC is contributing to the overall humoral immune response against IP administered antigens.

## Materials and Methods

### Virus

Salmonid alphavirus subtype 3 (SAV3) was provided by Øystein Evensen (Norwegian University of Life Sciences, Norway) and had been isolated from the heart tissue of Atlantic salmon with clinical signs of PD and identified as SAV3 by sequencing (25). The virus was propagated in CHH-1 cells (26) (Sigma), derived from the heart tissue of chum salmon (*Oncorhynchus keta*), in L-15 medium with 10 U/mL penicillin, 10 μg/mL streptomycin, and 5% FBS at 15°C. Virus titer was measured in the cell line CHSE-214 [Chinook salmon (*Oncorhynchus tshawytscha*) embryo cells] (26) using the procedure described previously (27) and calculated according to the TCID_50_ method (28).

### Fish and SAV3 Infection

Atlantic salmon (*Salmo salar*) of the strain Aquagen standard were used in two separate infection experiments with SAV3 performed at Tromsø Aquaculture Research Station (Tromsø, Norway). Before the start of each of the experiments, pre-smolts (∼30 g) were supplied with fresh water at 10°C, 24 h of light, fed commercial dry feed (Skretting, Norway) until satiation for seven weeks, and had then reached an average weight of 61.5 and 67.0 g, respectively. The experiments were then organized as described below. After an initial sampling of 10 fish, the fish were randomly allocated into two tanks. Individual fish in the infection tank were injected IP with 100 μL 10^5^ TCID_50_ SAV3 diluted in phosphate buffer saline (PBS) while fish in the control tank were injected IP with 100 μL PBS. For both experiments, fish were supplied with fresh water at 10°C and 24 h of light. The two experiments were terminated at 2 or 12 weeks post-infection (wpi), respectively. Feed was withheld for 24 h prior to all samplings. Blood and tissues were sampled from both groups at different time points after infection, as described below (*n* = 6–10). Prior to any invasive operation, fish were anaesthetized (40 μg/mL) or overdosed (80 μg/mL) with benzocaine (ACD Pharmaceuticals, Norway). The experiments were approved by the Norwegian Food Safety Authority (ID 11258 and 13827).

### Cell Isolation

Prior to sampling, the fish were bled by exsanguination from the caudal vein. The PerC was opened through the mid-ventral line and cells were collected by three rounds of lavage each with one mL of ice-cold PBS containing 2% fetal bovine serum (FBS) and 20 U/mL heparin. The lavage was mixed with 2 mL of ice-cold transport medium (L-15 medium with 10 U/mL penicillin, 10 μg/mL streptomycin, 2% FBS) and kept on ice. Peritoneal washes with visible blood contamination were discarded. HK and spleen were harvested from the same fish and homogenized using 100 μm cell strainers (Falcon). Leukocytes were isolated by layering PerC lavage or tissue homogenates on 25/54% discontinuous Percoll gradients (GE Healthcare) and centrifugation at 400×*g* for 40 min at 4°C (29). After collecting cells at the gradient interface, cells were washed twice in L-15 medium, counted, and kept on ice until used in downstream applications.

### Enumeration of Total IgM ASC by ELISpot

ELISpot assay was used to enumerate the total *ex vivo* IgM-secreting B cells from PerC, HK, and spleen. The assay was established by determining the optimal concentration of the coating antibody, detection antibody and streptavidin-HRP conjugate that gave distinct spots with minimal background staining. In addition, different concentrations of cells from each tissue were analyzed to determine the linear range of the assay (Supplementary Figure 1). MSIPS4510 plates (Merck Millipore) were activated with 35% ethanol before washing four times with PBS and coated overnight with 15 μg/mL of purified anti-trout IgM mAb [IgF1-18 (6-1-18)] (30). After four PBS washes, plates were blocked for 90 min at room temperature (RT) using L-15 with 2% bovine serum albumin (BSA) (Sigma). After four washes, 25,000 cells from either PerC, HK, or spleen were seeded in triplicate in 100 μL L-15 with 10 U/mL penicillin, 10 μg/mL streptomycin, and 5% FBS. Plates were incubated at 14°C for 48 h before washing five times with PBS containing 0.1% Tween 20 (wash buffer). For spot detection, 1.5 μg/mL biotinylated purified anti-trout IgM mAb [IgF1-18 (6-1-18)] (biotinylated using EZ-Link NHS-PEG solid phase biotinylation kit, Thermo Fisher Scientific) was added and incubated for 90 min at RT. After four washes, wells were incubated at RT with streptavidin-HRP (1:500) (Mabtech) for one hour. Spots were developed using TMB substrate (100 μL/well, Mabtech) for 10 min at RT in the dark, excessively washed with tap water and air-dried overnight. Wells with no cells (NCC) or with no detection biotinylated antibody (DAC) were included as controls on each plate. The plates were scanned digitally using ImmunoSpot image acquisition software and spot counts were determined automatically by C.T.L software (both from CTL). The IgM ASC frequency (*z*) was defined as the number of spots per 25,000 cells. The total number of ASC (*x*) in each tissue was calculated based on the frequency of ASC (*z*) and the total leukocyte count (*y*) for each tissue as follows: *x* = *y* × (*z*/25000).

### Flow Cytometry Analysis

The percentage of IgM^+^ B cells in the PerC, HK, and spleen was analyzed by flow cytometry as described previously (31). Immediately following isolation, 1 × 10^5^ cells were washed once in PBS with 0.5% BSA and stained with anti-trout IgM mAb [IgF1-18 (6-1-18); 1:200] for 30 min at 4°C. After two washes, cells were incubated for 20 min with isotype specific secondary Ab (IgG1-RPE; 1:400 dilution; Jackson ImmunoResearch), and FVD780 (1 μL/mL, Fixable Viability Dye eFluor 780, Invitrogen). After two washes, cells were resuspended in 100 μL fixation buffers (4% paraformaldehyde) and placed at 4°C overnight. Cells were washed twice and resuspended in 200 μL of PBS with 0.5% BSA and analyzed on a LSRFortessa analyzer (BD biosciences), while data analyses were done in FlowJo version 10 (Tree Star). FACSAria III (BD biosciences) was used to sort fresh unfixed granular cells (SSC^*high*^) from PerC into RNAprotect Cell Reagent. After dead cells (FVD780^+^) and doublets (FSC-A vs FSC-W) exclusion, IgM^+^ cells were gated on the whole leukocyte population or the lymphocyte gate (Supplementary Figure 2).

### RT-qPCR Analysis

Detection and relative quantification of SAV3 RNA in serum and heart samples was performed by RT-qPCR using primers for nsP1 (32). Secreted (sIgM) and membrane bound (mIgM) IgM (31) transcripts were analyzed from the same heart samples. For serum, virus RNA was extracted from 140 μL using QIAamp viral RNA kit and maximum RNA input was used in the cDNA synthesis reaction (12 μL) as recommended by the manufacturer (Qiagen). Heart tissue was homogenized in RLT buffer using TissueLyser II (Qiagen) and subsequently treated with proteinase K (Applied Biosystems) for 10 min at 55°C. Total RNA from sorted granular cells was harvested the same way as the heart tissue, omitting the proteinase K step. Total RNA from heart and granular cells was isolated using RNeasy Min kit (Qiagen) according to the manufacturer’s protocol. Six hundred nanogram RNA was reverse transcribed using QuantiTect Reverse Transcription Kit (Qiagen) according to the manufacturer’s protocol. PCR was run using SYBR green master mix (Applied Biosystems) in 20 μL reactions with 5 μL of 1:5 or 1:10 diluted cDNA from serum or tissues, respectively. Samples were analyzed on a 7500 Fast Real-Time PCR system (Applied Biosystems) with an initial denaturation of 20 s at 95°C and 40 cycles of 3 s at 95°C and 30 s at 60°C. Samples were run in duplicate and the EF1aB gene was used as an endogenous control. Relative expression was calculated by the 2^–ΔCt^ method (33), while fold changes in virus RNA was calculated by the 2^–ΔΔCt^ method (34). Primers used in this study are presented in Table 1.

**TABLE 1:** Primers used for RT-qPCR.

**Oligo name**	**Sequence**	**PCR efficiency**	**Accession number**
EF1aB	Fw: TGCCCCTCCAGGATGTCTAC	1.98	BG933897
	Rv: CACGGCCCACAGGTACTG		
SAV3 nsP1	Fw: CCGGCCCTGAACCAGTT	2.00	AY604235
	Rv: GTAGCCAAGTGGGAGAAAGCT		
mIgM	Fw: CCTACAAGAGGGAGACCGA	1.73	S48658
	Rv: GATGAAGGTGAAGGCTGTTTT		
sIgM	Fw: CTACAAGAGGGAGACCGGAG	2.04	BT060420
	Rv: AGGGTCACCGTATTATCACTAGTTT		
MCSF-R	Fw: CACCAGTAACCCTAACCACTTC	2.00	NM_001171807.1
	Rv: GACCTGCTTGTCCTGCATTA		
MPO	Fw: CGAAACACGACCTTCAACAAC	2.00	BT072012.1
	Rv: AACTCGCTATCGTTCACTACAC		

### Detection of Anti-SAV3 E2 Antibody Response in Serum by ELISA

Recombinant SAV3 E2 protein was used for coating ELISA plates. The preparation of the E2 protein is described elsewhere (35). Briefly, 96 well Maxisorp plates (Thermo Fisher Scientific) were coated overnight at 4°C with recombinant E2 protein (200 ng/well) and subsequently blocked with a protein free blocking buffer (Thermo Fisher Scientific) before incubation with 1:80 diluted individual sera samples in duplicate. Following overnight incubation at 4°C, wells were washed four times (PBS containing 0.05% Tween-20). Mouse anti-trout IgM mAb (IgF1-18 (6-1-18), 1:500) and HRP conjugated goat anti-mouse (Bio-Rad, 1:1500) were added sequentially and incubated at RT each for 1 h. Plates were developed for 30 min in the dark with OPD substrate (Sigma). Optical densities were measured immediately at 450 nm on a VersaMax microplate reader (Molecular devices).

### Measuring SAV Neutralizing Activity in Serum

Sera collected 3, 6, and 9 wpi were analyzed for SAV neutralizing activity. This was performed by Agri-Food and Biosciences Institute, Belfast, Ireland using an immunoperoxidase-based virus neutralization (VN) assay as described elsewhere (36). Briefly, sera were diluted 1/10, 1/15, 1/20, and 1/40 and pre-incubated with virus (100 TCID_50_ salmon pancreas disease virus strain F93-125) before inoculation on CHSE-214 cells. After three days, cells were fixed and stained with mAb 2D9 (1:8000), raised against the SPDV strain F93-125 (37, 38). Plates were examined microscopically and wells with specific viral staining were scored as negative. Neutralizing antibody titers were calculated as the reciprocal of the highest dilution giving specific viral staining. To rule out complement-mediated neutralization activity, sera from infected fish were heat inactivated at 43°C for 45 min and tested as described above.

### Total IgM Protein in Serum

Sera collected before (3 wpi) and after (6 wpi) the induction of the anti-SAV E2 antibody response were analyzed to determine the total IgM content by western blot. Total serum protein was quantified using a Micro BCA assay (Thermo Fisher Scientific) and 0.5 μg was loaded onto 4–12% gradient NuPAGE Novex Bis-Tris gels after denaturation with a 5 μL LDS buffer (4×) at 70°C for 10 min. Samples were subjected to SDS-polyacrylamide gel electrophoresis with a 1× MOPS buffer for 50 min at 200 V and 120 mA (Invitrogen). MagicMark^TM^ XP and SeeBlue Plus 2 pre-stained were used for molecular weight estimation (Invitrogen). Protein was blotted onto a polyvinylidene difluoride (PVDF) membrane, blocked and incubated overnight with anti-trout IgM mAb (IgF1-18 (6-1-18); 1:200). After four washes, the membrane was incubated with HRP conjugated anti-mouse (1:5000 dilution; Santa Cruz Biotechnology) for 1 h and developed using SuperSignal West Femto Trial Kit (Thermo Fisher Scientific) and a KODAK Image Station 4000MM Digital Imaging System. Band intensities (arbitrary unit, AU) were determined by subtracting the background signal from the visualized IgM band of ∼70 kDa.

### Statistical Analysis

Statistical analyses were done in GraphPad Prism version 5 or SPSS version 24. Statistical analyses on steady state IgM^+^ B cells and ASC from PerC, HK, and spleen were performed using two-tailed one-way ANOVA followed by a Bonferroni *post hoc* test when the F-statistic indicated a significant difference in the mean. A Mann–Whitney U test was used to analyze differences in IgM^+^ cells and ASC between control and infected fish at each sampling point. The Spearman correlation coefficient, r, was used to measure correlation between ASC count, serum antibody responses, and virus RNA in the heart. Differences were considered statistically significant at *p* < 0.05 and strength of significance is indicated by the number of asterisks (^∗^); where^∗^*p* < 0.05, ^∗∗^*p* < 0.01 and ^∗∗∗^*p* < 0.001.

## Results

### Steady State Head Kidney Harbors the Majority of IgM Secreting Cells in Atlantic Salmon

**FIGURE 1:**
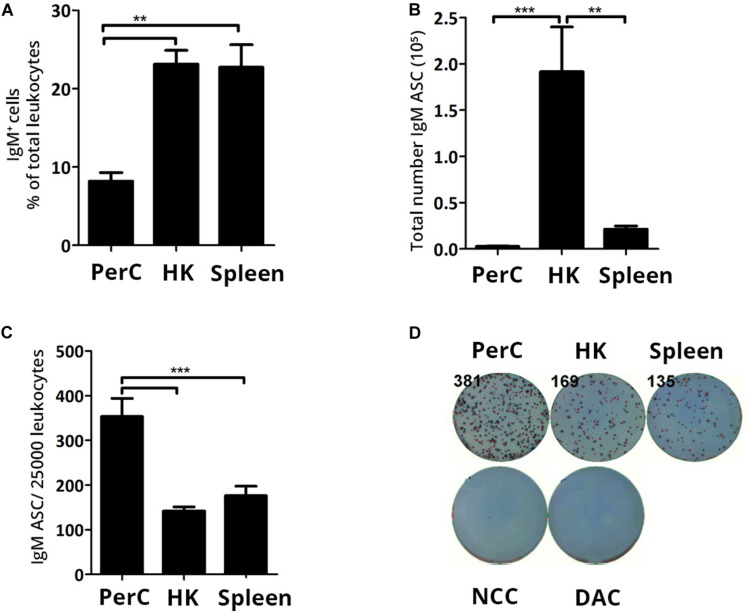
In steady state Atlantic salmon head kidney (HK) harbors the highest IgM ASC numbers of the three sites. Presence of B cells and IgM ASC were analyzed from naïve Atlantic salmon **(A)** IgM^+^ B cell frequency of total leukocytes was analyzed by flow cytometry. **(B)** Total IgM ASC or **(C)** IgM ASC per 25,000 leukocytes in PerC, HK, and spleen were analyzed by ELISpot. **(D)** Representative ELISpot wells. Statistical significance between tissues/sites are indicated by brackets. Asterisks indicate strength of significance: **p < 0.01 and ***p < 0.001. Data present mean + SEM from at least five individuals (*n* ≥ 5). NCC-No cell control; DAC, detection biotinylated antibody control.

Characterizing the resident B cell populations in naïve Atlantic salmon lays the groundwork for evaluating the effects infectious challenge triggers on the humoral immune response. In the current study, flow cytometry analysis showed a frequency of ∼23% IgM^+^ cells in naïve HK and spleen leukocytes. The frequency of IgM^+^ cells in the PerC (∼8%) was significantly lower (*p* < 0.01) compared to HK and spleen (Figure 1A). ELISpot was used to enumerate the total number of IgM ASC at the same sites and HK harbored approximately a 10-fold higher IgM ASC count (mean ± SEM = 1.9 × 10^5^ ± 47468) than spleen (mean ± SEM = 2.1 × 10^4^ ± 3497.2) (Figure 1B). PerC possessed the lowest total number of ASC (mean ± SEM = 2742 ± 542), although it had more than a two-fold higher ASC frequency compared to the systemic sites (*p* < 0.01) (Figure 1C). In summary, a small population of IgM ASC comprising 0.6–1.4% of total leukocytes was found to reside in the systemic lymphoid tissues and PerC, respectively, of naïve Atlantic salmon with the total IgM ASC count being highest in HK.

### IgM^+^ B Cells and IgM Secreting Cells Take Part in the Early PerC Responses to SAV3

**FIGURE 2:**
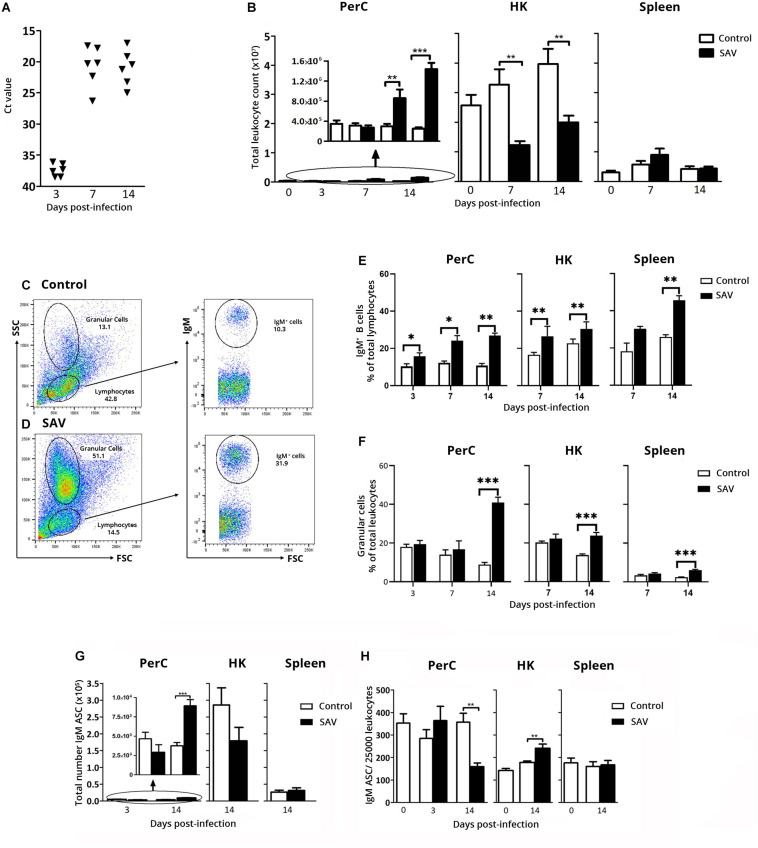
Early PerC response to IP SAV3 infection in Atlantic salmon involves IgM^+^ B cells and IgM ASC. **(A)** RNA levels of SAV3 nsP1 in serum samples, presented as Ct values. **(B)** Total leukocyte count in PerC, HK, and spleen. Representative flow cytometry cytograms showing the gating of IgM^+^ B cells, and granular cells in the PerC of **(C)** control and **(D)** SAV3 infected fish. Flow cytometry analysis of IgM^+^ B cells presented as **(E)** frequency of total lymphocytes and **(F)** Percentage granular cells of total leukocytes. IgM ASC analyzed by ELISpot in the PerC, HK, and spleen from control and SAV3 infected fish presented as **(G)** total IgM ASC per site or **(H)** IgM ASC frequency per 25,000 leukocytes. Statistical significance between control and SAV3 groups are indicated by brackets. Asterisks indicate strength of significance: **p < 0.01 and ***p < 0.001. For all analysis, data present mean + SEM from at least six individuals (*n* ≥ 5).

Local PerC responses induced by IP stimulation have been studied in teleost focusing on the very early responses of the myeloid and lymphoid cell populations (11, 12, 39). In this study one of the goals was to understand the PerC IgM^+^ B cell response in the long term course of a viral infection. In the first experiment, PerC and systemic IgM^+^ B cell responses, were assessed for up to 2 weeks after IP SAV3 infection. Establishment of infection was confirmed by detection of virus RNA in serum using RT-qPCR. At 3 days post-infection (dpi), trace amounts of SAV3 nsp1 RNA (Ct ≤ 36.4) was detected in sera samples from four out of six fish, while at 8 and 14 dpi, virus RNA was detected in all examined fish (Figure 2A).

The effect of the SAV3 infection on the total leukocyte count in the PerC was evident by a 2.9- and 5.7-fold increase at 7 and 14 dpi, respectively, while a decrease in total leukocyte count was observed in the HK at the same time points (2.6- and 2.0-fold decrease, respectively) (Figure 2B). This could suggest a virus-induced migration of cells from the primary lymphoid organ (HK) to various sites affected by SAV3 including the PerC. In spleen, the total leukocyte count was not significantly affected by the infection (Figure 2B).

Flow cytometry analyses of the total leukocyte population in PerC, HK, and spleen showed a general increase in the frequency of IgM^+^ B cells from 3 dpi in the infected fish compared to the controls (Figure 2E). At 14 dpi, in infected fish IgM B^+^ cells comprised 27, 31, and 46% of cells within the lymphocyte gate in PerC, HK, and spleen, respectively, corresponding to a 2.5, 1.3, and 1.8 fold increase (*p* < 0.05) relative to controls (Figure 2E).

To expand the understanding of virus-induced B cell responses, the total number of IgM ASC at the three immune sites was determined using ELISpot. Although the number of time points analyzed were limited, a significant increase in total IgM ASC (2.4-fold compared to control) was observed in the PerC at 14 dpi (mean ± SEM = 8920 ± 809) (*p* < 0.001) (Figure 2G). In HK, a 1.6-fold decrease in total IgM ASC occurred at 14 dpi from 2.86 × 10^5^ in the control to 1.79 × 10^5^ in the infected fish. No changes were apparent in the spleen. Despite this increase in total IgM ASC in PerC, the IgM ASC frequency was significantly lower (*p* < 0.01) in the PerC of infected fish compared to controls at 14 dpi, decreasing from 1.41% in the control to 0.62% in the infected fish of the total cell population (Figure 2H). This is likely accounted for by the change in the PerC leukocyte population as shown by flow cytometry (Figures 2C,D), where the frequency of FSC^*low*^SSC^*high*^ granular cells increased 4.7 times in infected fish (41% of leukocyte population) 14 dpi (Figure 2F), which correspondingly decreased the frequency of IgM ASC (Figure 2H). Note that at the same time point this granular cell population increased 1.7 times in the HK (24% of the leukocyte population) and 2.5-fold in spleen (6% of the leukocyte population) (Figure 2F). Gene expression analysis showed a 27.4 times higher expression of myeloperoxidase (MPO, Ct 20.6), a putative granulocyte marker gene, than macrophage colony stimulating factor receptor (M-CSFR, Ct 25.3) on FACS sorted granular cells from the PerC of infected fish (Supplementary Figure 3).

### The PerC of Atlantic Salmon Mounts Prolonged IgM^+^ B Cell and IgM Secreting Cell Responses

**FIGURE 3:**
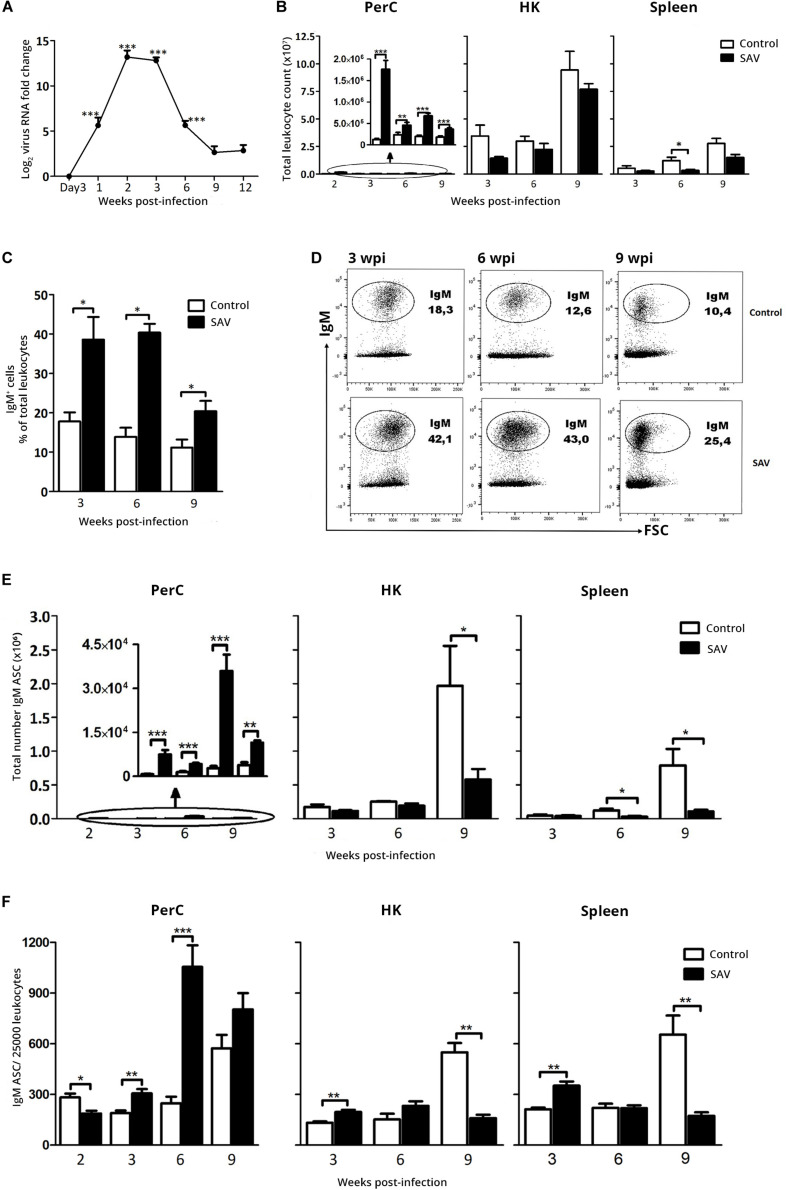
IP SAV3 infection promotes long-term B cell responses in the PerC of Atlantic salmon. **(A)** SAV3 nsP1 RNA levels in the heart from three days to 12 weeks post infection were analyzed by RT-qPCR. Presented as mean + SEM of fold-change (2^–ΔΔ*Ct*^ method) (*n* = 8). **(B)** Total leukocyte count for PerC, HK, and spleen from control and SAV3 infected fish (*n* = 5–10). **(C)** IgM^+^ B cell frequency in PerC was analyzed by Flow cytometry presented as mean + SEM (*n* = 3–6). **(D)** A representative cytogram showing the frequency of IgM^+^ cells in the PerC of control and SAV3 infected fish. IgM ASC was analyzed by ELISpot in the PerC, HK, and spleen from control and SAV3 infected fish (*n* = 5–10) presented as **(E)** total IgM ASC per site or **(F)** IgM ASC frequency per 25,000 leukocytes. Statistical significance between control and SAV3 groups are indicated by brackets. Asterisks indicate strength of significance: * *p* < 0.05, **p < 0.01, and ***p < 0.001. The graph embedded in “PerC” shows the difference in total leukocyte and IgM ASC count over time on a different y-axis scale.

The virus-induced changes observed at 14 dpi triggered our interest on how these responses would evolve over a longer period of time, an issue that has not been previously addressed in most teleost fish species including Atlantic salmon. A similar IP infection experiment with SAV3 was set up, focusing the majority of analyses on later time points, from 3 to 9 wpi. As for the first experiment, no mortality was recorded, while SAV3 infection was confirmed and virus RNA in the heart peaked at 2 to 3 wpi (Ct mean ± SEM = 25.8 ± 1.9) with a subsequent decline until 9 wpi, where it stabilized (Figure 3A). At 9 wpi, virus RNA was undetectable in two of 10 fish analyzed and was just within the threshold of detection in the remaining fish (Ct value = 33.1–36.6). The peak virus RNA in heart at 2 wpi paralleled the peak total leukocyte count (Figure 3A) and severity of gross pathological conditions in the PerC, which included accumulation of exudate and hyperemia of the cavity wall (not shown). In addition, a significantly lower body weight was observed in the infected fish compared to the controls from 3 wpi onward (Supplementary Figure 4), a common clinical sign of fish developing PD (18).

The total PerC leukocyte count in infected fish at 2 wpi corresponded to the levels in the early response experiment (Figures 2B, 3B). This response was sustained, although at lower levels, from 3 to 9 wpi with a 2- to 2.5-fold increase compared to the control (Figure 3B). No significant changes in total leukocyte counts were observed in HK of infected fish, while a significant (3.7-fold) decrease was apparent in the spleen of infected fish at 6 wpi (Figure 3B). Further, flow cytometry showed an increase in the frequency of IgM^+^ cells in the PerC of infected fish (*p* < 0.05) which sustained until the last sampling at 9 wpi (Figures 3C,D). It peaked at 3–6 wpi at which the IgM^+^ cells accounted for close to 40% of the total PerC population.

ELISpot revealed a significant increase in the total IgM ASC count in the PerC of infected fish compared to the control (Figure 3E). At 6 wpi the total PerC IgM ASC response in the infected fish peaked with a 13-fold increase (Mean ± SEM = 35891 ± 5638) (Figure 3E). In the systemic lymphoid tissues, HK and spleen, a reduction in the total IgM ASC count was observed for the infected fish from 6 to 9 wpi (Figure 3E). Except at 2 wpi, the infection resulted in an increased frequency of IgM ASC in PerC (Figure 3F), showing that the ASC response at this site is more predominant at later time points.

Of note is also the general increase in both total leukocyte and IgM ASC counts in HK and spleen of the control group with increasing age of the fish (Figures 3B,E). This change was particularly distinctive from 6 to 9 wpi. However, the PerC of uninfected fish was not affected to the same extent. The control fish did not show any signs of disease that could explain the change and were negative for the presence of SAV3 (Ct ≥ 38.5).

### SAV3 Infection Induces Transcription of IgM in the Heart

**FIGURE 4:**
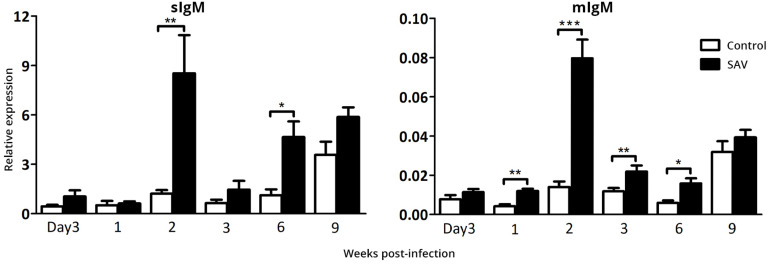
Biphasic expression of IgM transcripts in the heart from Atlantic salmon infected with SAV3. The expression of secreted (sIgM) and membrane (mIgM) IgM were determined by RT-qPCR in the heart from control and SAV3 infected fish and relative expression was calculated by the 2^–ΔCt^ method. Data present mean + SEM from at least eight individuals (*n* = 8–10). Statistical significance between control and SAV3 groups is indicated by brackets. Asterisks indicate strength of significance.

The heart is a main target tissue for SAV3 replication in Atlantic salmon making it an interesting tissue for studying local B cell responses. Salmonid alphavirus subtype 3 presence was confirmed in heart (Figure 3A) and both membrane (mIgM) and secreted (sIgM) IgM transcript levels peaked at 2 wpi (Figure 4), which mirrored the peak in the viral RNA level (Figure 3A). A 7.1 and 5.7-times fold increase of sIgM and mIgM transcripts, respectively, were found in infected fish compared to the controls at 2 wpi, suggesting either virus-induced trafficking to or local expansion of B cells (Figure 4). However, due to differences in basal expression (sIgM mean Ct = 19.8; mIgM mean Ct = 26.1), sIgM was 107 times higher transcribed in heart compared to mIgM. A second phase of IgM expression was evident from 3 to 9 wpi, with increasing levels in the controls but also significantly higher (*p* < 0.05) virus-induced expression of both sIgM and mIgM in 6 wpi.

### The Virus Specific Serum Ab Responses Correlate With the IgM Secreting Cell Count in the PerC

**FIGURE 5:**
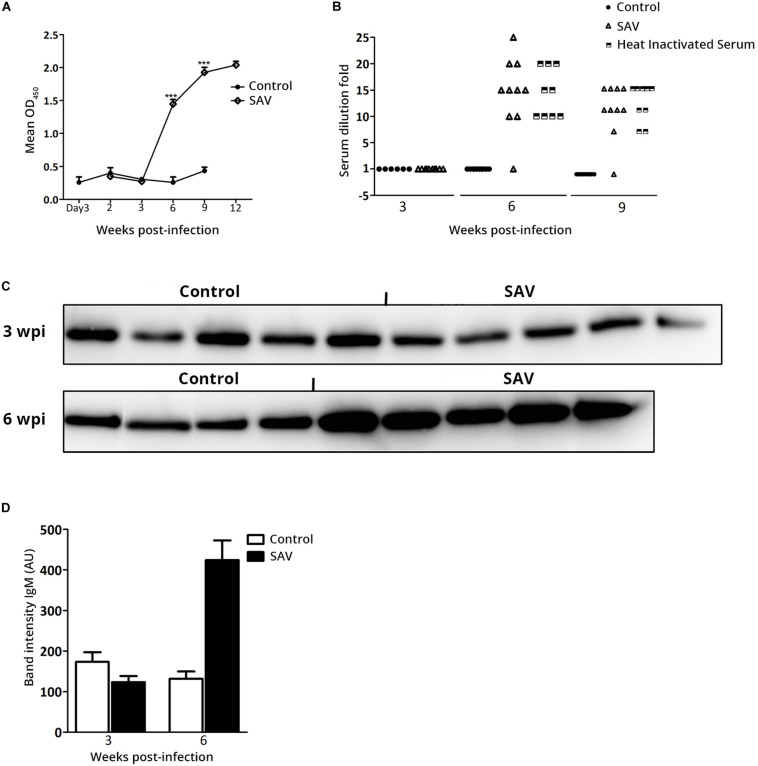
Atlantic salmon mounts antibody-mediated anti-viral response against SAV3 infection. **(A)** Anti-SAV3 E2 antibody response in serum from control and SAV3 infected Atlantic salmon were analyzed using ELISA (data presented as mean OD_450_ + SEM; *n* = 6 for control and *n* = 10 for infected). **(B)** Virus neutralizing titers in serum from control and SAV3 infected Atlantic salmon before and after heat inactivation (Scatter plot of individual values; *n* = 6 for control and 10 for infected). **(C)** Representative Western blots showing total IgM protein in individual serum samples at 3 and 6 wpi in control (*n* = 4) and SAV3 infected Atlantic salmon (*n* = 5). **(D)** Mean band intensity of IgM (AU, arbitrary units) determined by subtracting the background noise from the visualized bands from blots shown in panel **(C)**.

The specific Ab response directed against SAV E2 and the virus neutralizing activity were analyzed using ELISA and VN assay. Neither anti-E2 Ab response nor virus neutralizing activity was detected until 6 wpi, where 10/10 fish were seroconverted based on ELISA (Figure 5A) and virus neutralizing activity was detected in 9/10 fish (Figure 5B). A significant increase in anti-E2 Ab response was observed from 6 to 12 wpi (Figure 5A). The neutralizing activity of the sera samples was not affected by heat treatment, indicating that it had been mediated by virus specific Abs and not by complement (Figure 5B). The anti-E2 Ab response (OD value) and neutralizing titer correlated negatively with virus RNA in the heart (*p* < 0.001), while they were positively correlated with the total IgM ASC count in the PerC (*p* < 0.001). The total serum IgM protein level was measured by western blot and the result was significantly higher (*p* < 0.001) for infected fish compared to the controls at 6 wpi (Figures 5C,D).

## Discussion

Distinct features of teleost adaptive immunity, such as absence of follicular structures and germinal centers and the dependence on un-switched IgM responses make these species, such as Atlantic salmon, useful comparative models of lower vertebrate immunity. Vaccination induces specific Ab responses that protect Atlantic salmon against subsequent infection with the same pathogen; however, the nature of the adaptive B cell response is mostly unknown in this species. Gaining insight in this field is highly relevant for today’s aquaculture industry where viral diseases, such as PD caused by SAV, persist to be a major problem despite widespread vaccination. The majority of viral vaccines in fish are inactivated virus particles injected IP. Here we used IP infection with live SAV3 as a model to delineate Atlantic salmon B cell responses with special focus on the local PerC response versus the response in systemic lymphoid tissues. PerC cells are easily harvested making it a suitable model for studying local versus systemic immune responses. We also considered it more likely to obtain a robust immune response using live virus versus an inactivated viral antigen, making it easier to establish the ELIspot and FACS assays in a proper manner.

As an initial step, the presence of IgM^+^ cells and IgM ASC were analyzed in naive, healthy Atlantic salmon and compared between the PerC and systemic sites, HK and spleen. Flow cytometry analysis displayed, as expected, high frequency of IgM^+^ cells in the systemic tissues (∼23%) (Figure 1A). However, in HK the frequency was higher than previously reported (9%) by us (31). The immune system of fish is highly dependent on their physiological conditions [reviewed in Refs. (40, 41)], which might account for this discrepancy as we used different size fish in the two studies. Our results further confirm the presence of IgM^+^ cells in steady state teleost PerC, which previously has been shown in other fish species (7, 10, 16, 42). In the present study, 8% of PerC leukocytes were IgM^+^ cells, while a rainbow trout study reported a higher frequency (32–42%) (7), maybe influenced by a 5 to 6°C higher water temperature in this study or due to intraspecies variation in leukocyte composition.

IgM ASC were present in all examined sites, with the PerC harboring a 2-fold higher IgM ASC frequency than HK and spleen (Figure 1C). Similar IgM ASC distribution was also found in the same size but a different batch of Atlantic salmon (data not shown) showing reproducibility of the results. In rainbow trout, the presence of spontaneously ASC has been shown in HK and spleen by ELISpot (43). To our knowledge, PerC IgM ASC has not been previously reported in teleosts. In mice, bone marrow (BM) and spleen are the major sites where spontaneous IgM-secreting B cells are located, corresponding with the presence of IgM ASC in Atlantic salmon HK and spleen. Mice PerC B cells may also be IgM secretors, but they secrete much lower amounts of IgM per cell compared to BM and spleen B cells, observed as very small spots in the ELISpot assay (44). Spontaneous secretion of natural IgM is a characteristic of B-1 B cells and hence, spleen and BM B-1 B cells account for the majority of serum IgM in naïve, pathogen free mice (45). The functional role of the IgM spontaneously secreted by PerC B-1 cells may be a local and not a systemic response [reviewed in Ref. (46)]. In our studies, no difference in spot size was apparent between the PerC and systemic tissues indicating secretion of similar amounts of IgM (data not shown). Serum of naïve, healthy Atlantic salmon contains ∼1 mg/ml of IgM [reviewed in Ref. (47)], however, which B cells at which site(s) accounted for the spontaneous serum IgM in Atlantic salmon, or teleosts in general, has not been looked into. Lack of experimental methods to study Atlantic salmon B cell characteristics renders further functional studies in this direction challenging.

The early response experiment showed a prominent virus-induced increase in total leukocyte counts in the PerC from 7 dpi and onward. In HK a corresponding decrease in total leukocyte counts was evident, suggesting migration of cells from the HK to the PerC and possibly to other tissues within the first two weeks of infection. Due to the poor availability of leukocyte specific markers in Atlantic salmon, our possibilities to characterize these mobilized cell populations were limited. However, FSC vs SSC distribution of cells from PerC, HK, and spleen revealed an increased presence of a cell population with a more granular morphology than lymphocytes (Figures 2D,F). Gene expression analysis on these PerC granular cells revealed a higher expression of MPO over M-CSFR, suggesting that granulocytes dominated this population (Supplementary Figure 3). As in higher vertebrates, MPO is an enzymatic marker for neutrophils also in lower vertebrates (48, 49). Whether MPO is restricted to neutrophils, or if other myeloid cells in Atlantic salmon also express MPO has, to our knowledge, not been studied. Neutrophils are the first responders to inflammation with reservoirs in teleost HK. Upon IP immune challenge, teleost neutrophils migrate rapidly from HK to the PerC and normally resolves inflammation within 72 h [reviewed in Ref. (50)]. However this has been mostly studied using strong inducers of acute inflammation, such as bacterial infection. Characterizing the SAV-induced granular cell population observed in Atlantic salmon PerC, HK, and spleen is an interesting direction for future viral infection experiments.

While the SAV3-induced increase in total PerC IgM ASC count started at 2 wpi, the virus-dependent increase in total and specific serum IgM did not appear until 6 wpi (Figure 5). This indicates that the IgM secreted by the PerC ASC functions locally at the site of SAV3 administration from 2 to 6 wpi and is possibly not transferred into the circulation during this period. A reason for this could be a low secretion rate of the ASC or a low IgM half-life at this early stage of the B cell response to SAV3. In rainbow trout these parameters have been reported to increase over time as the B cell response matures [reviewed in Ref. (51)]. Our data do not support the above claim as the spot size in the ELISpot assay, which is an estimate of IgM secretion rate, from the early (2 wpi) and late (9 wpi) samplings did not differ significantly (data not shown). Our PerC IgM ASC data should also be seen in relation to gene expression data obtained from the heart (Figure 4), revealing a pattern similar to that observed in the PerC. At 2 wpi, at the peak of SAV3 RNA levels, virus-induced IgM expression was markedly dominated by sIgM over mIgM transcripts, indicating a local ASC response also here. Whether these early IgM responses appearing in the PerC and heart, prior to the serum IgM response represents production of low affinity polyreactive IgM (natural Ab) or highly specific IgM is presently not known. However, this apparently local IgM response in the heart may have a role in the observed decrease in viral load from 2 to 3 wpi (Figure 3A). In mice, activation of B cells in the PerC and pericardial cavity induces secretion of polyreactive IgM that acts locally [reviewed in Ref. (52)]. Clusters of stromal and immune cells in visceral adipose tissues, called milky spots or fat-associated lymphoid clusters, are important niches for the maintenance and activation of these B cell populations [reviewed in (52)]. In rainbow trout, visceral adipose tissue in the PerC has been shown to contain B cells and retain antigen (15, 53, 54), suggesting a similar role of this tissue in teleosts. These structures have not been studied in Atlantic salmon, and whether the SAV3 used in our study can initiate an infection and/or be retained here is not known.

One question to consider is how the observed B cell response after IP SAV3 infection is initiated. It is known that teleost B cells are phagocytic (55, 56), and in rainbow trout ∼30–40% of IgM^+^ cells recovered from PerC had engulfed beads or bacteria after IP injection (56). A virus infection in rainbow trout IgM^+^ B cells upregulates expression of MHCII and co-stimulatory molecules suggesting a virus induced APC phenotype (57). Further, a study in zebrafish has demonstrated that teleost B cells could non-specifically ingest both particulate and soluble antigens and act as initiating APCs to prime CD4^+^ T cells(58). The specific consequences of this for Ab production remains to be elucidated. In mice this phagocytic and antigen presenting role of B cells is specifically linked to the PerC B-1 cell population and not to conventional B-2 cells (59). A study in Atlantic salmon revealed that IP injected ovalbumin is endocytosed by MHCII^+^ cells in the periphery (PerC) and that these cells, over a period of 14 days, accumulate in the HK (60), suggesting a continuous trafficking of leukocytes between the peripheral and systemic immune sites. Detailed studies on this cell population indicate that they are professional APCs supporting the role HK has as a major secondary lymphoid organ. In the same study, the MHCII^+^/IgM^+^ cell population (B cells) appeared to have a minor role. In our study, both of the above described priming mechanisms can possibly have occurred directly in PerC.

A striking result of the present study was the prolonged presence (up to 9 wpi) of a virus-induced ASC response in PerC (Figures 3E,F). To our knowledge, B cell responses in the PerC of such duration have not been previously reported for teleosts. A question to ask is how the SAV3-induced ASC in the PerC are maintained and what their role is. Whether there was a productive SAV3 infection in PerC leukocytes or surrounding tissues was not the scope of this study, but trace amounts of SAV3 RNA was found in PerC leukocytes at 6 wpi (Ct ≥ 34, data not shown) indicating a prolonged presence here. It is possible that virus-persistence leads to local prolonged activation of B cells into ASC or that the ASC might migrate from systemic sites, given the decrease in total ASC number and ASC frequency in the HK and spleen (Figures 3B). BAFF, a B cell survival factor, has recently been characterized in rainbow trout and been shown to promote survival of rainbow trout PerC ASC (17), but whether virus-persistence can induce BAFF expression and thereby support PerC ASC survival in the long-term is not known.

The current study monitored the PerC ASC response for up to 9 wpi and the response was waning from 6 to 9 wpi. Whether the virus-induced ASC response at this site further declines back to basal levels in control fish or is sustained for a longer period is presently not known. Studies in rainbow trout has shown a peak antigen specific ASC response in blood, spleen, and HK 8 weeks after immunization. From 10 weeks and onward the response decreased in blood and spleen, while in HK the specific ASC was sustained with characteristics of long-lived plasma cells (43). The SAV3-specific ASC response was not quantified in our study, and whether the virus-specific serum Ab titers are linked to the presence of SAV antigen specific ASC in the PerC or in the systemic tissues will be a topic for future studies.

Of note is also the ∼2- to 3-fold increase in ASC frequency in the control group from 6 to 9 weeks (Figure 3E), present at all the three sites. We are not aware of any methodological limitations that could have caused this. To check the reproducibility of this data, however, more fish were subjected to the same analysis three days later and the results were similar, supporting the biological relevance of this change in ASC frequency. Although the control fish gained significantly higher weight compared to the infected fish from 3 wpi, the weight difference at 9 wpi was striking (Supplementary Figure 4). Whether changes in ASC counts can be linked to this substantial growth or other physiological changes, such as naturally or stress-induced fluctuating hormone levels, is not known [reviewed in Ref. (41)]. Interestingly, the SAV-infection appeared to suppress this age-related increase in IgM ASCs observed in the control fish (Figures 3E,F). The mechanisms behind this is not known and warrants further investigation.

In this study, a SAV3 E2 specific serum Ab response was detected from 6 to 12 wpi corresponding to the detection of the SAV-neutralizing Ab response (Figures 5A,B). Others have previously shown induction of neutralizing Ab responses against SAV (23) (21, 22), however, only recently was an ELISA established using E2 as the target antigen (35, 61), supporting our data and confirming the reactivity of Atlantic salmon B cells to SAV3 E2. The fact that sera samples retained similar neutralizing titers after heat inactivation excluded involvement of complement and further confirmed the Ab mediated anti-viral role described above. At 9 and 12 wpi, low levels of SAV3 persisted in the heart (Ct > 33), indicating the induction of non-sterile immunity. Although the longevity of the specific Ab response against SAV in Atlantic salmon is not known, the persistence of the virus at low level can possibly be involved in maintaining virus specific Abs in the long-term.

In summary, meeting the demand for more efficacious viral vaccines requires further efforts on understanding the orchestration of B cell responses. This is to our knowledge the first report comparing the presence of ASC and IgM^+^ B cells in the PerC of an untreated healthy teleost species. Our findings showed uneven distribution of ASC and IgM^+^ B cells between the PerC and systemic sites at steady state, warranting further investigation to clarify whether this is due to possible lineage differences or the presence of other factors that sequester ASC in the PerC. This work presents a prolonged IgM^+^ B cell and ASC response in the PerC of Atlantic salmon infected with SAV3. The local PerC ASC response against SAV3 correlates positively with the virus specific Ab and neutralizing responses in serum, suggesting further that it may contribute to the overall humoral response against the virus. These findings call for further studies to clarify the precise anatomical location and cellular constellation of a putative secondary immune tissue in Atlantic salmon PerC, which could widen our understanding of teleost humoral immunity.

## Data Availability Statement

The raw data supporting the conclusions of this article will be made available by the authors, without undue reservation.

## Ethics Statement

The animal study was reviewed and approved by Norwegian Food Safety Authority Norway.

## Author Contributions

SJ, MP, HT, and MS acquired the data and performed the data analysis. JJ and IJ designed the *in vivo* experiments, supervised the data analysis, and obtained the funding. SJ and IJ wrote the manuscript, JJ and MP revised it. All authors performed the *in vivo* experiments and subsequent *in vitro* cell work and read the manuscript and approved it.

## Conflict of Interest

The authors declare that the research was conducted in the absence of any commercial or financial relationships that could be construed as a potential conflict of interest.
