# Supplementary material for: Salmonid Alphavirus Subtype 3 Induces Prolonged Local B Cell Responses in Atlantic Salmon (Salmo salar) After Intraperitoneal Infection
Source: Front Immunol. 2020 Sep 10;11:1682. doi: 10.3389/fimmu.2020.01682 (PMC7511533; doi:10.3389/fimmu.2020.01682)
Supplement: Supplementary file 1 [file Data_Sheet_1.PDF]

Supplementary Material

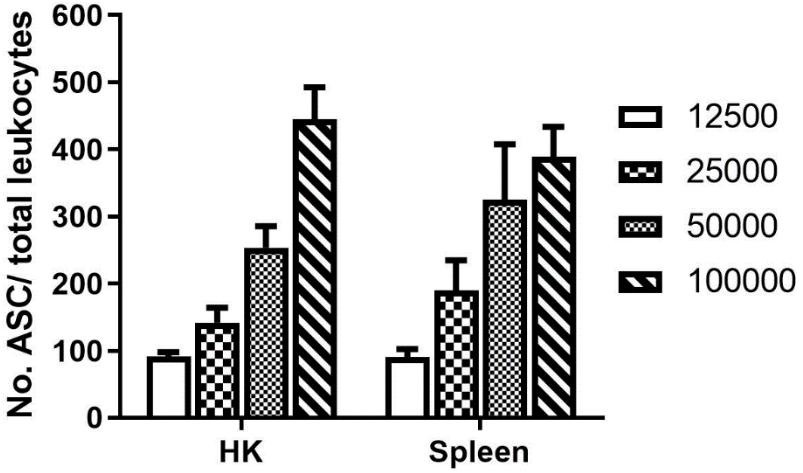

Supplementary Figure 1. Total IgM ELISpot for different seeding densities of leukocytes. Leukocytes were isolated from HK and spleen and two-fold dilutions of cells ($1x10^5$, $5x10^4$, $2.5x10^4$, $1.25x10^4$) were seeded into triplicate wells of ELISpot plates. Total IgM ELISpot assay was performed as described in the Materials and Methods section. ELISpot numbers for each seeding density are shown as average of five to three individuals.

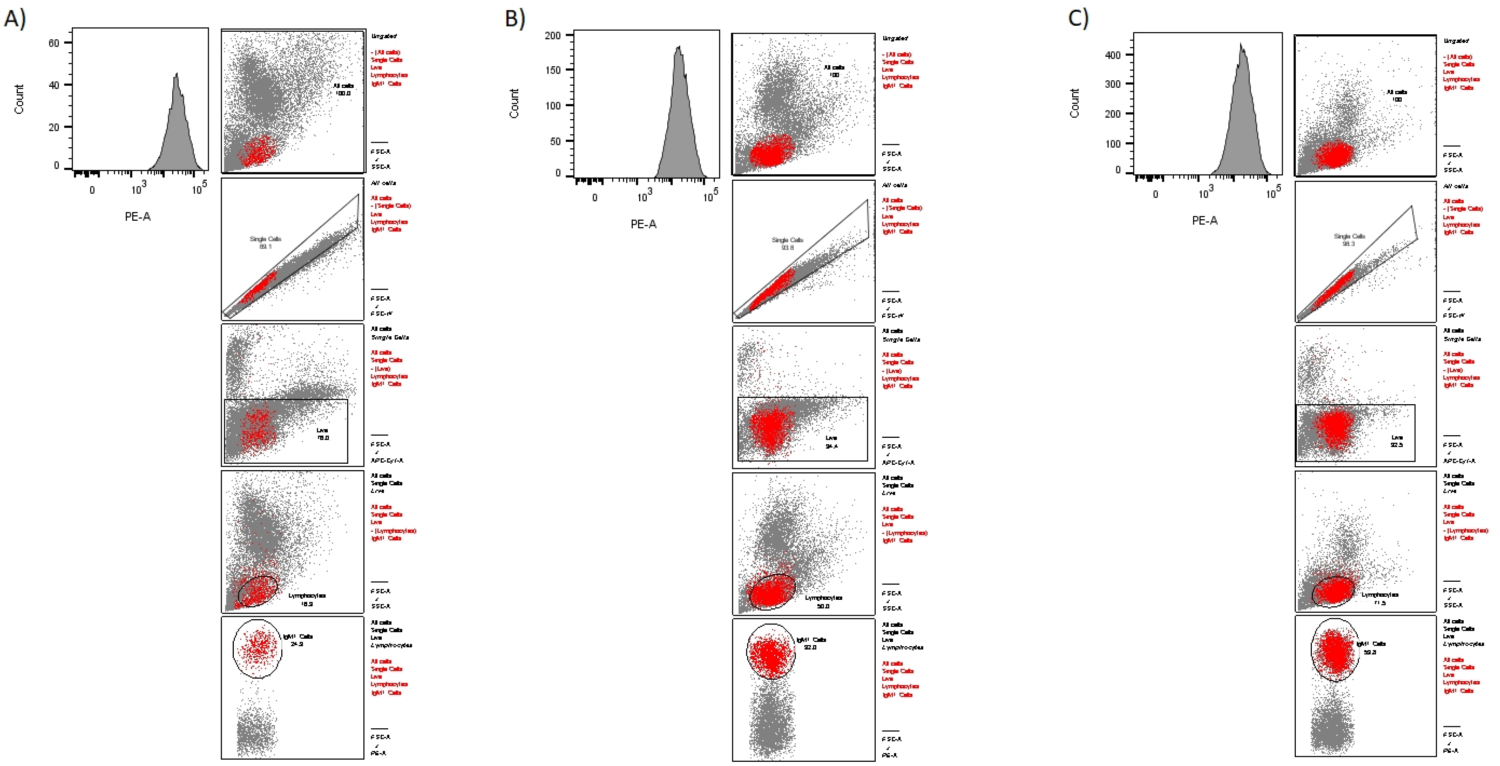

Supplementary Figure 2. Representative flow cytometry analysis showing the gating strategy for IgM^{+} cells. After doublet (FSC-A vs FSC-W) and dead cells (FVD780^{+}) exclusion, live cells were gated for further analysis. Based on the FSC-A vs SSC-A properties of the live single cells, lymphocyte gate was drawn. IgM^{+} B cells were then analyzed from the lymphocyte gate based on their specific staining characteristics (PE^{+}). The IgM^{+} cells gating hierarchy for the PerC (A), HK (B) and spleen (C) leukocytes was displayed using FlowJo's backgating option.

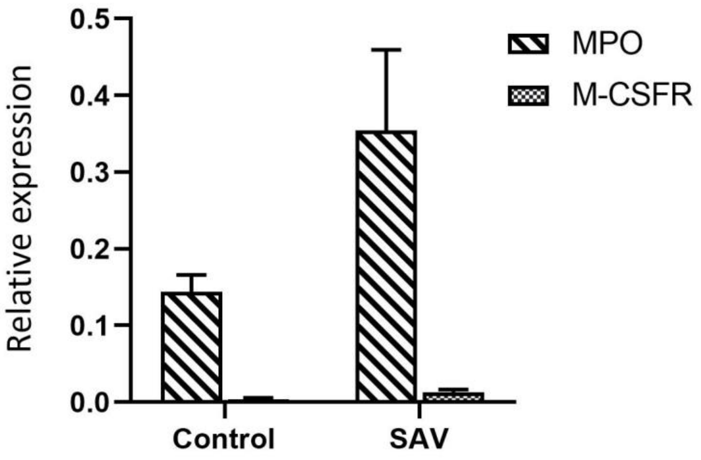

Supplementary Figure 3. Relative expression of granulocyte marker (MPO) and macrophage marker (M-CSFR) genes in sorted FSC^{low}SSC^{high} cells. PerC FSC^{low}SSC^{high} granular cells from control and SAV infected fish were FACS sorted and total RNA was extracted. RT-qPCR was used to quantify the expression of MPO and M-CSFR. Relative expression of MPO and MCSF-R was calculated using the $2^{-\Delta Ct}$ method. Data present mean + SEM from three individuals (n = 3).

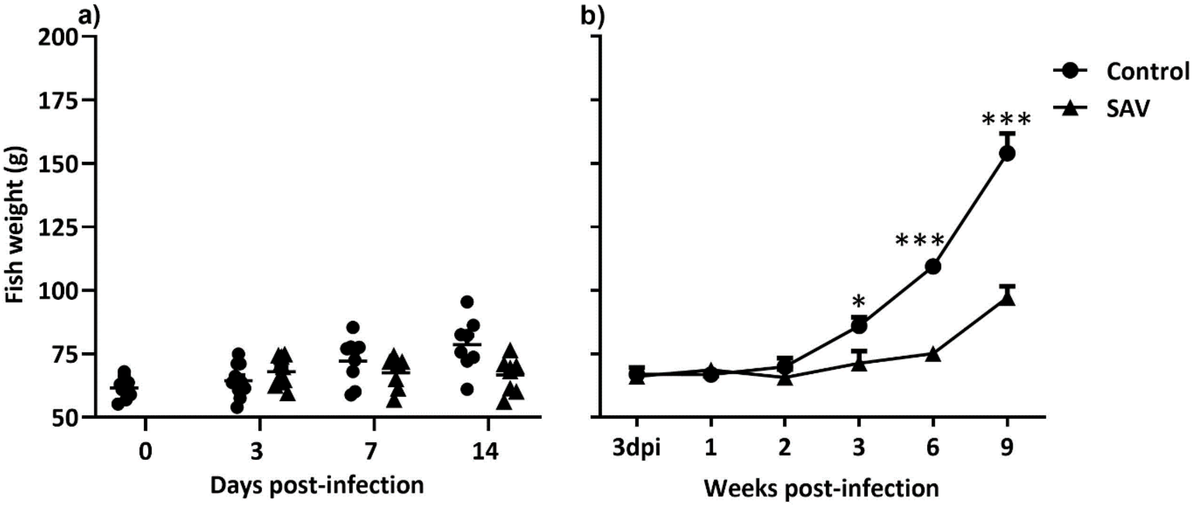

Supplementary Figure 4. SAV infection impairs fish growth over time. Body weight in grams from (a) the early response and (b) prolonged response experiment is presented as mean + SEM (n = 8 to 10). Asterisks indicate statistical significance between control and SAV groups, and the number of asterisks indicates strength of significance. dpi- days post-infection.
